# Relationships between Food Production Experience and the Behavior, Attitude, and Knowledge of Dietary Recommendations among Japanese Adults: A Cross-Sectional Study

**DOI:** 10.3390/nu14173639

**Published:** 2022-09-02

**Authors:** Daisuke Machida

**Affiliations:** Home Economics Education Course, Cooperative Faculty of Education, Gunma University, Aramaki 4–2, Maebashi 371-8510, Japan; machi@gunma-u.ac.jp; Tel.: +81-27-220-7344

**Keywords:** food production experience, agricultural experience, forestry experience, fishery experience, healthy diet, sustainable diet, dietary guideline, food and nutrition education, Japan

## Abstract

The Japanese Ministry of Agriculture, Forestry, and Fisheries proposed to educate more people in food production as one of the objectives of the Basic Plan for Food and Nutrition Education Promotion. This cross-sectional study aims to elucidate the relationship between food production experience (agricultural, forestry, and fishery experiences) and the behavior, attitude, and knowledge of dietary recommendations among Japanese adults. This study was conducted using data obtained from the “Survey on Dietary Habits and Agriculture, Forestry, and Fishery Experiences (2019)”. This survey was conducted by mailing paper questionnaires to respondents aged 20–69 years living in 17 regions across Japan. The independent variable was food production experience. The dependent variables were each of the 13 goals of the dietary guidelines in Japan, the total score for adherence to the 13 items (low scores indicate good adherence), adherence to a Japanese-style diet, and four items on the attitude and knowledge of dietary recommendations. I also examined the association between the point in life the food production experience occurred and the above outcomes. The data obtained from 3461 participants aged 20 to 69 years were used for multivariate logistic and linear regression analyses. Food production experience was positively associated with an improved behavior, attitude, and knowledge of dietary recommendations among adults in Japan. The study found a strong relationship with food production experiences in adulthood.

## 1. Introduction

Food production experiences (agricultural, forestry, and fishery experiences) are positively associated with dietary behavior, awareness, and knowledge [1,2,3,4,5,6,7,8,9,10,11,12,13,14,15,16]. Several review articles suggest a positive association between food production experience and healthy-eating knowledge or positive attitudes toward fruits and vegetables [1,2,3,4]. Other reviews report positive associations between food production experience and dietary behavior, such as fruit and vegetable intake, balanced meal intake, and improved food access; however, the results are partially inconsistent for fruit and vegetable intake [1,2,3,4,5,6,7,8,9,10,11,12,13]. Additionally, several Japanese studies report the associations between food production experience and dietary behavior, awareness, and knowledge [14,15,16]. Most of the food production experiences involve the production of crops through gardening or other means; however, some include the experience of making processed foods from crops harvested on the farm [14,15,16]. Most of the studies treated school or community gardening as a form of exposure/intervention [14,15,16]. In addition, no study considered forestry or fishing experiences as standalone exposures/interventions [14,15,16]. As with studies in other countries, Japanese studies have reported inconsistent results on the association between fruit and vegetable intake, although reports of positive associations predominate [14,16]. Moreover, the associations are not the only factors contributing to health, such as an increased preference for and intake of fruits and vegetables or increased diversity in food intake [14,15]. Scholars also observed positive associations with dietary awareness related to environmental conservation, such as a sense of gratitude for food and less food waste [14,16]. Thus, food production experiences are related to multifaceted dietary behavior, awareness, and knowledge. Because of this background, the Japanese Ministry of Agriculture, Forestry, and Fisheries proposes that increasing people’s food production experience is one of the objectives of the Basic Plan for Food and Nutrition Education Promotion [17].

However, no studies have determined whether differences exist in dietary behavior, awareness, and knowledge during adulthood and beyond according to the life stage at which the food production experience occurs. The associations between food production experience and dietary behavior, awareness, and knowledge have been examined at various life stages [1,2,3,4,5,6,7,8,9,10,11,12,13,14,15,16]. Many studies on children have used gardens in kindergartens and schools as interventions and exposures. Many studies on adults and beyond have used community, allotment, and urban gardens as interventions and exposures [1,2,3,4,5,6,7,8,9,10,11,12,13,14,15,16]. In terms of design, many of these studies are cross-sectional or interventional ones that evaluate short-term effects [1,2,3,4,5,6,7,8,9,10,11,12,13,14,15,16]. In other words, no studies have determined whether food production experiences in childhood exert long-term effects on eating habits in adulthood and beyond, or whether only food production experiences during adulthood and beyond are relevant to eating habits in adulthood and beyond.

This cross-sectional study determines the associations between food production experiences and the behavior, attitude, and knowledge of dietary recommendations in Japan. Then, this study examines whether differences exist in behavior, attitude, and knowledge in adulthood according to the life stage at which food production was experienced.

## 2. Materials and Methods

### 2.1. Data

The data were derived from the Survey on Dietary Habits and Agriculture, Forestry, and Fishery Experiences 2019 (Ministry of Agriculture, Forestry and Fisheries) from the Social Science Japan Data Archive, Center for Social Research and Data Archive [18,19]. The objective of the survey was to confirm the level of recognition of the Japanese Dietary Balance Guide, the level of implementation of dietary guidelines for Japanese, and the proportion of respondents who participated in food production experiences throughout Japan. Moreover, the survey intended to provide basic data for future policy development. This survey was conducted using paper questionnaires mailed to respondents aged between 20 and 69 years and living in 17 regions across Japan [18]. The survey was conducted by dividing Japan into four areas (Tokyo area, Kinki area, regional cities, and small cities/counties); setting the percentage of the total sample for each of the four areas; and then allocating the sample to each area by sex, age, and household composition according to the population composition ratio of the 2015 National Population Census [18]. Random sampling was conducted according to the allocation performed by survey monitors from Rakuten Insight, Inc. (Tokyo, Japan) [18].

Anonymous data were obtained from 3645 individuals, out of which data from 3461 individuals with complete responses were included for analysis. This study was conducted using anonymous information from a previously completed survey and per the ethical guidelines for life science and medical research involving human subjects in Japan [20]. This report followed the STROBE Statement [21].

### 2.2. Variables

#### 2.2.1. Independent Variables

The study employed the status of food production experience as the independent variable (with and without experience [ref.]). The survey asked, “Have you or has anyone in your family ever participated in a food production experience?” [18]. If a respondent answered “yes”, the respondent was asked whether they had participated in the experience [18]. Only those respondents who had themselves participated in food production were treated as “with experience” in this study.

The life stage at which the respondents had experienced food production (elementary school ages or younger, junior-high-school ages or older (less than 20 years old), 20 years old or older, and without experience [ref.]) was used as another independent variable. The respondents were asked for the life stage at which they had experienced food production in multiple questions with nine categories (younger than elementary school age; elementary school age; junior-high-school age; high-school age; 10s other than above; in their 20s, 30s, 40s, and 50s or older). Then, these categories were aggregated into the three categories listed above based on the distribution of responses and station-in-life fit [18].

#### 2.2.2. Outcomes

I used the behavior, attitude, and knowledge of dietary recommendations as outcomes. Adherence to dietary guidelines for Japanese and Japanese-style diets was used for dietary behavior. For dietary attitude and knowledge, the following items were used: attitude to food and nutrition education, knowledge about the Japanese Dietary Balance Guide, knowledge of dietary guidelines for Japanese, and knowledge of Japanese-style diet.

*Adherence to dietary guidelines for Japanese.* The main feature of the dietary guidelines for the Japanese is that they were created with a broad perspective on the entire dietary system, which ranges from food production and distribution to dining and health [22]. The guidelines emphasize improving quality of life; focusing on a well-balanced diet; and considering a stable supply of food, the food culture, and the environment [22]. The dietary guidelines for Japanese consist of the following preferred practices: (1) enjoy your meals; (2) establish a healthy rhythm by keeping regular hours for meals; (3) maintain appropriate weight through adequate exercise and well-balanced meals; (4) eat well-balanced meals with staple food, as well as main and side dishes; (5) eat enough grains, such as rice and other cereals; (6) combine vegetables, fruits, milk products, beans, and fish in your diet; (7) avoid too much salt, and attention should be paid to the quality and quantity of fat ingested; (8) take advantage of the Japanese dietary culture and local food products; preserve local dishes; (9) conserve food resources and practice dietary habits that minimize leftovers and food waste; and (10) develop your understanding of food and review your dietary life [22]. Respondents to the Survey on Dietary Habits and Agriculture, Forestry, and Fishery Experiences 2019 were asked to respond to the items of the dietary guidelines, which were rated using a four-point Likert-type scale (1 = *usually*; 2 = *often*; 3 = *seldom*; 4 = *never*). Notably, practices 6 and 7 could not be captured using one question. Thus, their items were presented as follows: (6a) ensure vitamin, mineral, and fiber intakes by consumption of vegetables and fruit daily; (6b) maintain calcium intake from milk, dairy products, green and yellow vegetables, beans, and small fish; (7a) avoid food and dishes with a high salt content; (7b) consume balanced proportions of fats from animals, vegetables, and fish; and (7c) establish a habit of checking nutrition fact labels when selecting food and eating out [18]. In this study, the total score for the 13 items (13–52 points: the lower the score, the higher the adherence) and each item (two categories: *usually*/*often* or *seldom*/*never* [ref.]) were used for analyses. The reliability of the total score was acceptable (Cronbach’s α = 0.857).

*Adherence to a Japanese-style diet*. The following question focused on this: “Are you practicing a Japanese-style diet?”. This item was rated using the four-point Likert-type scale. Two categories (*usually*/*often* or *seldom*/*never* [ref.]) were used for analyses. The question described the Japanese-style diet as one that consists of rice and various side dishes, such as fish, meat, milk, dairy products, vegetables, seaweed, legumes, fruits, and green tea. It is assembled within a few days to a week and can be combined with retort food, frozen food, and eating out. The Japanese-style diet intake is inversely associated with the risk of functional disability, dementia, and mortality due to cardiovascular diseases [23,24,25].

*Attitude and knowledge*. For attitude toward food and nutrition education, the participants were asked about their interest in food and nutrition education using four categories: interested, somewhat interested, somewhat uninterested, and uninterested. Two categories (*interested/somewhat interested* or *somewhat uninterested/uninterested* [ref.]) were used for analyses. This item is commonly used to measure the interest of the Japanese people in food and nutrition education [26]. In terms of knowledge about the Japanese Dietary Balance Guide, dietary guidelines for Japanese, and Japanese-style diet, the participants were asked about the degree of their knowledge about each item using three responses: “I know the item including its contents”, “I have heard of it at least by name”, and “I do not know it”. Responses to the first two questions were summarized as “known”. Two values, namely, *known* and *unknown* [ref.], were used for analyses. Adherence to the Japanese Dietary Balance Guide reportedly reduces the risk of mortality [27,28].

#### 2.2.3. Basic Characteristics

The following basic characteristics were used for analyses: sex (women; men), age (20s, 30s, 40s, 50s, and 60s), marriage status (not married; married), household structure (living alone; married couple; parents and children; grandparents, parents, and children; other).

### 2.3. Analyses

First, the analysis was conducted on food production experience as the independent variable. Multiple regression analysis was used when the total score for dietary guideline adherence was used as the outcome variable. The other outcome variables were composed of binary data. Thus, binary logistic regression analysis was used.

Multiple regression and binary logistic regression analyses were then conducted to determine the life stages at which the respondents experienced food production as the independent variable. Here, sensitivity analyses that included only those aged 50 and older were conducted because young adults would have less opportunity to experience food production after the age of 20 years than middle-aged adults would have had.

For both analyses, crude and adjusted models using the basic characteristics (age was not used in sensitivity analysis) as covariates were created, and regression coefficients (B) or odds ratios (ORs) and 95% confidence intervals (95%CI) were calculated.

SPSS Statistics 28.0 (IBM Japan, Ltd., Tokyo, Japan) was used for analyses with a significance level of 5% (two-tailed test).

## 3. Results

Table 1 presents the responses according to food production experience.

Table 2 describes the relationships between food production experience and the behavior, attitude, and knowledge of dietary recommendations. The total score for dietary guideline adherence was lower for people with experience than those without experience in food production (adjusted model: B = − 1.496, *p* < 0.001). In other words, adherence was better among those who experienced food production. The trend was similar for many items when the analysis was conducted for each item (adjusted models: OR: 1.044–1.869, *p*: <0.001–0.554). However, salt and calcium intakes indicated no significant associations. The adherence to a Japanese-style diet was also positively related to food production experience (OR = 2.176, *p* < 0.001). In addition, attitude to food and nutrition education and knowledge of the Dietary Balance Guide, dietary guidelines, and Japanese-style diet were positively related to food production experiences (adjusted models: OR: 1.468–2.046, all *p*-values < 0.001).

Table 3 illustrates the relationships between the life stage at which food production was experienced and the behavior, attitude, and knowledge of dietary recommendations. For the life stages, 798, 304, and 624 experienced food production during their elementary school ages or younger, junior-high-school ages or older, and at 20 years old or older, respectively. A total of 2187 lacked this experience. For the total score for dietary guideline adherence, only those with experience at 20 years old or older exhibited a significantly positive relationship (adjusted model: B = −1.556, *p* < 0.001). Food production experience at this life stage also exerted significant relationships with each item of the dietary guidelines (except for enjoyment, salt intake, and fat intake), adherence to the Japanese-style diet, attitude to food and nutrition education, and knowledge of Dietary Balance Guide, dietary guidelines, and Japanese-style diet. Food production experience during elementary school ages or younger displayed significantly positive relationships with adherence to Japanese-style diet, attitude to food and nutrition education, and knowledge of Dietary Balance Guide, dietary guidelines, and Japanese-style diet. However, only a few relationships with dietary guideline adherence (traditional and sustainable diets were positively significant; other items and total scores were non-significant). Additionally, food production experience during junior-high-school ages or older exhibited no relationship with nearly all outcomes. Per each item in the dietary guideline adherence, food production experience during junior-high-school ages or older pointed to significant relationships with enjoyment, dietary rhythm, and sustainable diet. For attitude and knowledge, the knowledge of the Japanese-style diet was significantly related to food production experience during this life stage as well.

Table 4 illustrates the results of the sensitivity analyses. For subjects in their 50s and 60s, the B and OR for the life stage at which food production experience occurred was very similar to the B and OR for behavior, attitude, and knowledge of dietary recommendations. Food production experience at an age of 20 years was significantly positively associated with the total score for dietary guideline adherence, 7 out of 13 items for dietary guideline adherence, adherence to a Japanese-style diet, and 3 out of 4 items for attitude and knowledge of dietary recommendations. The difference from the overall analysis was that the OR of experience in elementary school age or younger on “Practice sustainable dietary habits” and “Knowledge of dietary guidelines” was closer to 1.

## 4. Discussion

This study examined the association between food production experiences and the behavior, attitude, and knowledge of dietary recommendations. Furthermore, the study examined whether differences exist in behavior, attitude, and knowledge of dietary recommendations in adulthood and beyond according to the life stage at which food production was experienced. The results reveal that an adherence to the dietary guidelines for Japanese and Japanese-style diets; attitude to food and nutrition education; and knowledge of the Japanese Dietary Balance Guide, dietary guidelines for Japanese and Japanese-style diets were higher in people with food production experiences. In addition, food production experiences at 20 years old or older displayed significant positive associations with behavior, attitude, and knowledge of dietary recommendations. Furthermore, food production experiences during elementary school or younger were almost not associated with dietary behaviors. However, a significantly positive association between attitude and knowledge of dietary recommendations was noted. Moreover, food production experiences during junior-high-school ages or older exhibited almost no association with behavior, attitude, and knowledge of dietary recommendations. This study strengthened the evidence of the association between food production experiences and behavior, attitude, and knowledge of dietary recommendations among Japanese adults. Furthermore, this study was the first to examine the relationship between previous experiences with food production and behavior, attitude, and knowledge of dietary recommendations in adulthood and beyond, which provide new insights into related studies globally.

Positive associations between food production experiences and behavior, awareness, and knowledge are widely known [1,2,3,4,5,6,7,8,9,10,11,12,13,14,15,16]. However, no previous studies used dietary guidelines for Japanese as an outcome; thus, the current study added new knowledge to this field. The dietary guidelines for the Japanese were developed not only to recommend a healthy diet, but also to promote the continuation of traditional diets and sustainability [20]. Therefore, this study inferred that food production experience was positively related to a desirable diet in terms of health, tradition, and sustainability. Previous Japanese studies demonstrated the positive associations between food production experiences and dietary awareness related to environmental conservation and knowledge of traditional foods, which is consistent with the present results [14,16]. This study used a comprehensive item on traditional food preservation and sustainable diets. Previous studies have used indicators, such as knowledge of specific local traditional foods, food waste behavior, and a sense of gratitude for food [14,16]. Future studies should scrutinize these relationships using more specific and systematic indicators.

The results examined, according to the life stage at which food production was experienced, reveal a significant association between food production experience and behavior, attitude, and knowledge of dietary recommendations during adulthood and beyond. Previous cross-sectional studies on Japanese subjects reported positive associations during adulthood and beyond, consistent with the current results [14,15,16]. In addition, these studies did not adjust for food production experiences in childhood [14,15,16]. The present study found that even after adjusting for food production experiences in childhood, food production experiences in adulthood were positively related to dietary behaviors, attitudes, and knowledge. The association is not simply causal in that food production experiences could lead to desirable dietary behavior, attitude, and knowledge, including a reverse causal relationship in those individuals with high levels of dietary awareness frequently under food production experiences. The study inferred that food production experiences during adulthood interact with dietary awareness.

In addition, food production experience during childhood was almost non-significantly associated with dietary behaviors during adulthood. However, food production experiences during elementary school ages and younger were significantly associated with attitude and knowledge of dietary recommendations during adulthood and beyond. Therefore, food production experiences during elementary school or younger may influence dietary attitudes and knowledge during adulthood and beyond. Furthermore, sustainable dietary behaviors in adulthood and beyond were positively associated with food production experiences in elementary school ages or younger and junior-high-school ages or older. In other words, food production experiences during childhood may exert long-term effects on sustainable dietary behaviors that may continue into adulthood and beyond. In this regard, the previous studies pointed out that awareness of environmental conservation can be reinforced by preventing the “extinction of experience” and “shifting the baseline syndrome” [29,30]. The first expression denotes less direct contact with the natural environment [29]. The second expression pertains to a change in accepted norms for the natural environment condition due to the extinction of experience [30]. Presumably, direct contact with nature during experiences with food production could prevent these conditions. Food production experiences during childhood may play a role in environmental conservation from the dietary perspective as a method for avoiding these problems. Given the growing interest in the sustainable diet in recent years, food production experiences may be an important factor in the future [31]. However, in the sensitivity analysis for subjects over 50 years old, there was no longer any association between experience at elementary school age or younger in the categories of “Practice sustainable dietary habits” and “Knowledge of dietary guidelines”. The influence of food production experiences at elementary school age or younger may persist among young adults but not into middle age and older. More detailed studies will be needed in the future.

### Limitation

This study had several limitations. First, the subjects were Japanese; thus, caution should be exercised when generalizing the interpretation of the results to other countries and regions. Although many scholars worldwide have reported simple associations between food production experiences during adulthood and food, following up on the long-term effects of food production experiences during childhood in other countries and regions would be desirable. Moreover, all survey items were self-reported; thus, the possibility of recall bias was undeniable. Future research using objective indicators is needed. Furthermore, analyses were conducted using survey items commonly used in Japanese government statistics; as such, a debate may emerge about the validity and reliability of the survey items. Given that the data were obtained from a cross-sectional survey, further verification of the causal relationship between items found to be significantly related to the study is required.

## 5. Conclusions

Food production experience was positively associated with the behavior, attitude, and knowledge of dietary recommendations among Japanese adults. This association was particularly significant for food production experience at 20 years old or older. Food production experience during elementary school ages or younger was positively associated with attitude and knowledge of dietary recommendations. Sustainable dietary practices were associated with food production experiences during elementary school ages or younger, junior-high-school ages or older, and 20 years old or older. It was suggested that will be important to develop a social infrastructure that allows people to experience food production not only as children, but also as adults.

## Figures and Tables

**Table 1:** Responses based on food production experience.

	Food Production Experience			
	Without Experience		With Experience	
	n	%	n	%
	2187		1274	
*Sex*				
Women	1083	49.5	692	54.3
Men	1104	50.5	582	45.7
*Age*				
Their 20s	277	12.7	255	20.0
Their 30s	453	20.7	264	20.7
Their 40s	500	22.9	303	23.8
Their 50s	442	20.2	213	16.7
Their 60s	515	23.5	239	18.8
*Marriage*				
Not married	696	31.8	374	29.4
Married	1491	68.2	900	70.6
*Household structure*				
Living alone	339	15.5	185	14.5
Married couple	453	20.7	203	15.9
Parent(s) and child(ren)	1245	56.9	767	60.2
Grandparent (s), parent (s), and child (ren)	124	5.7	99	7.8
other	26	1.2	20	1.6
*Behavior*	M	SD	M	SD
Total score for dietary guideline adherence *	30.2	6.1	28.6	6.0
Items for adherence to dietary guidelines **	n	%	n	%
(1) Enjoy your meals	1719	78.6	1076	84.5
(2) Establish a healthy dietary rhythm	1523	69.6	943	74.0
(3) Maintain appropriate body weight	1205	55.1	749	58.8
(4) Eat a well-balanced meal	1472	67.3	916	71.9
(5) Eat enough grains	1785	81.6	1111	87.2
(6a) Eat enough fruit and vegetable	1319	60.3	856	67.2
(6b) Maintain calcium intake	1468	67.1	888	69.7
(7a) Reduce salt intake	1116	51.0	657	51.6
(7b) Consume fats appropriately	1258	57.5	798	62.6
(7c) Use nutritional fact labels	586	26.8	429	33.7
(8) Preserve traditional diets	688	31.5	574	45.1
(9) Practice sustainable dietary habits	1745	79.8	1107	86.9
(10) Develop your understanding of food	1109	50.7	768	60.3
Adherence to a Japanese-style diet	1212	55.4	910	71.4
*Attitude and knowledge*				
Attitude to food and nutrition education	1646	75.3	1110	87.1
Knowledge of Dietary Balance Guide	1182	54.0	866	68.0
Knowledge of dietary guidelines	651	29.8	522	41.0
Knowledge of Japanese-style diet	858	39.2	601	47.2

M: mean; SD: standard deviation. * 13–52 points: the lower the score, the higher the adherence. ** (1) Enjoy your meals; (2) establish a healthy rhythm by establishing regular times for meals; (3) maintain appropriate weight through adequate exercise and well-balanced meals; (4) eat well-balanced meals with staple food, as well as main and side dishes; (5) eat enough grains, such as rice and other cereals; (6a) ensure vitamin, mineral, and fiber intakes by consumption of vegetables and fruit on a daily basis; (6b) maintain calcium intake from milk, dairy products, green and yellow vegetables, beans, and small fish; (7a) avoid food and dishes with high salt content; (7b) consume a balanced intake of fats from animals, vegetables, and fish; (7c) establish a habit of checking nutrition fact labels when selecting food and eating out; (8) take advantage of the Japanese dietary culture and local food products, and preserve local dishes; (9) conserve food resources and practice dietary habits to minimize leftovers and food waste; and (10) develop your understanding of food and review your dietary life. The “n” and “%” of “Items for adherence to dietary guidelines”, “Adherence to Japanese-style diet”, and “Attitude and knowledge” represent positive responses (*usually/often*; *interested/somewhat interested*; *known*).

**Table 2:** Relationships between food production experience and behavior, attitude, and knowledge of dietary recommendations (N = 3461).

	Crude Models		Adjusted Models	
	B (95%CI)	*p*	B (95%CI)	*p*
*Behavior*				
Total score for dietary guideline adherence *	−1.547 (−1.964, −1.129)	<0.001	−1.496 (−1.901, −1.091)	<0.001
Each item of dietary guideline adherence **	OR (95%CI)		OR (95%CI)	
(1) Enjoy your meals	1.479 (1.232, 1.776)	<0.001	1.368 (1.131, 1.654)	0.001
(2) Establish a healthy dietary rhythm	1.242 (1.063, 1.450)	0.006	1.213 (1.032, 1.425)	0.019
(3) Maintain appropriate body weight	1.162 (1.010, 1.337)	0.035	1.205 (1.045, 1.391)	0.010
(4) Eat a well-balanced meal	1.242 (1.068, 1.445)	0.005	1.255 (1.069, 1.473)	0.005
(5) Eat enough grains	1.535 (1.260, 1.868)	<0.001	1.429 (1.170, 1.747)	<0.001
(6a) Eat enough fruit and vegetable	1.347 (1.165, 1.557)	<0.001	1.401 (1.205, 1.629)	<0.001
(6b) Maintain calcium intake	1.126 (0.970, 1.307)	0.117	1.148 (0.984, 1.339)	0.078
(7a) Reduce salt intake	1.021 (0.889, 1.173)	0.759	1.044 (0.903, 1.207)	0.554
(7b) Consume fats appropriately	1.238 (1.074, 1.426)	0.003	1.267 (1.094, 1.466)	0.001
(7c) Use nutritional fact labels	1.387 (1.194, 1.611)	<0.001	1.377 (1.182, 1.605)	<0.001
(8) Preserve traditional diets	1.786 (1.549, 2.060)	<0.001	1.869 (1.613, 2.166)	<0.001
(9) Practice sustainable dietary habits	1.679 (1.383, 2.037)	<0.001	1.747 (1.434, 2.127)	<0.001
(10) Develop your understanding of food	1.475 (1.282, 1.697)	<0.001	1.467 (1.270, 1.695)	<0.001
Adherence to a Japanese-style diet	2.011 (1.734, 2.331)	<0.001	2.176 (1.858, 2.548)	<0.001
*Attitude and knowledge*				
Attitude to food and nutrition education	2.224 (1.838, 2.691)	<0.001	2.046 (1.679, 2.493)	<0.001
Knowledge of Dietary Balance Guide	1.804 (1.561, 2.085)	<0.001	1.676 (1.440, 1.952)	<0.001
Knowledge of dietary guidelines	1.637 (1.417, 1.892)	<0.001	1.637 (1.412, 1.896)	<0.001
Knowledge of Japanese-style diet	1.383 (1.203, 1.590)	<0.001	1.468 (1.272, 1.695)	<0.001

B: regression coefficient (linear regression analysis); OR: odds ratio (logistic regression analysis); CI: confidence interval. Independent variable: food production experience (experienced, non-experienced [ref.]) * 13–52 points: the lower the score, the higher the adherence. ** (1) Enjoy your meals; (2) establish a healthy rhythm by establishing regular times for meals; (3) maintain appropriate weight through adequate exercise and well-balanced meals; (4) eat well-balanced meals with staple food, as well as main and side dishes; (5) eat enough grains, such as rice and other cereals; (6a) ensure vitamin, mineral, and fiber intakes by consumption of vegetables and fruit on a daily basis; (6b) maintain calcium intake from milk, dairy products, green and yellow vegetables, beans, and small fish; (7a) avoid food and dishes with a high salt content; (7b) consume a balanced intake of fats from animals, vegetables, and fish; (7c) establish a habit of checking nutrition fact labels when selecting food and eating out; (8) take advantage of the Japanese dietary culture and local food products, and preserve local dishes; (9) conserve food resources and practice dietary habits to minimize leftovers and food waste; and (10) develop your understanding of food and review your dietary life.

**Table 3:** Life stage at which food production experience occurred and behavior, attitude, and knowledge of dietary recommendations (N = 3461).

	Crude Models		Adjusted Models	
	B (95%CI)	*p*	B (95%CI)	*p*
*Behavior*				
Total score for dietary guideline adherence *				
Elementary school age or younger	−0.194 (−0.723, 0.334)	0.471	−0.458 (−0.979, 0.062)	0.085
Junior-high-school ages or older	−0.547 (−1.341, 0.246)	0.176	−0.684 (−1.450, 0.081)	0.080
20 years or older	−2.070 (−2.608, −1.532)	<0.001	−1.556 (−2.083, −1.028)	<0.001
Each item of dietary guidelines adherence **				
(1) Enjoy your meals	OR (95%CI)		OR (95%CI)	
Elementary school ages or younger	1.238 (0.981, 1.562)	0.072	1.170 (0.918, 1.492)	0.203
Junior-high-school ages or older	1.417 (0.966, 2.078)	0.074	1.528 (1.032, 2.263)	0.034
20 years old or older	1.286 (1.009, 1.638)	0.042	1.149 (0.892, 1.481)	0.279
(2) Establish a healthy dietary rhythm				
Elementary school ages or younger	0.798 (0.659, 0.966)	0.021	0.848 (0.695, 1.036)	0.109
Junior-high-school ages or older	1.362 (1.003, 1.849)	0.048	1.475 (1.078, 2.017)	0.015
20 years old or older	1.642 (1.325, 2.034)	<0.001	1.381 (1.105, 1.724)	0.004
(3) Maintain appropriate body weight				
Elementary school ages or younger	0.885 (0.741, 1.056)	0.177	0.979 (0.816, 1.175)	0.823
Junior-high-school ages or older	1.139 (0.871, 1.490)	0.339	1.128 (0.860, 1.480)	0.381
20 years old or older	1.406 (1.170, 1.690)	<0.001	1.331 (1.101, 1.608)	0.003
(4) Eat a well-balanced meal				
Elementary school ages or younger	0.868 (0.719, 1.049)	0.144	0.979 (0.802, 1.196)	0.842
Junior-high-school ages or older	0.936 (0.703, 1.245)	0.650	1.025 (0.761, 1.381)	0.867
20 years old or older	1.743 (1.414, 2.149)	<0.001	1.420 (1.139, 1.769)	0.002
(5) Eat enough grains				
Elementary school ages or younger	1.318 (1.024, 1.697)	0.032	1.152 (0.886, 1.497)	0.288
Junior-high-school ages or older	1.522 (0.993, 2.333)	0.054	1.538 (0.997, 2.372)	0.051
20 years old or older	1.284 (0.989, 1.667)	0.060	1.329 (1.018, 1.736)	0.036
(6a) Eat enough fruit and vegetable				
Elementary school ages or younger	0.957 (0.798, 1.148)	0.641	1.090 (0.902, 1.319)	0.370
Junior-high-school ages or older	1.037 (0.786, 1.368)	0.796	1.102 (0.829, 1.464)	0.502
20 years old or older	1.580 (1.301, 1.918)	<0.001	1.365 (1.116, 1.670)	0.002
(6b) Maintain calcium intake				
Elementary school ages or younger	0.899 (0.746, 1.085)	0.270	1.000 (0.824, 1.214)	0.994
Junior-high-school ages or older	1.019 (0.767, 1.355)	0.893	1.065 (0.797, 1.423)	0.667
20 years old or older	1.403 (1.148, 1.713)	0.001	1.230 (1.000, 1.513)	0.050
(7a) Reduce salt intake				
Elementary school ages or younger	0.914 (0.767, 1.090)	0.321	1.063 (0.883, 1.279)	0.517
Junior-high-school ages or older	0.946 (0.727, 1.232)	0.685	0.984 (0.749, 1.293)	0.912
20 years old or older	1.334 (1.114, 1.597)	0.002	1.131 (0.936, 1.366)	0.201
(7b) Consume fats appropriately				
Elementary school ages or younger	0.972 (0.813, 1.163)	0.763	1.089 (0.904, 1.311)	0.368
Junior-high-school ages or older	1.124 (0.856, 1.476)	0.397	1.157 (0.876, 1.528)	0.302
20 years old or older	1.356 (1.125, 1.634)	0.001	1.206 (0.994, 1.463)	0.056
(7c) Use nutrition fact labels				
Elementary school ages or younger	1.174 (0.971, 1.419)	0.097	1.161 (0.953, 1.414)	0.137
Junior-high-school ages or older	1.045 (0.789, 1.384)	0.756	1.044 (0.785, 1.389)	0.763
20 years old or older	1.405 (1.164, 1.697)	<0.001	1.396 (1.149, 1.696)	0.001
(8) Preserve traditional diets				
Elementary school ages or younger	1.135 (0.946, 1.363)	0.172	1.290 (1.065, 1.562)	0.009
Junior-high-school ages or older	1.015 (0.773, 1.333)	0.913	1.046 (0.791, 1.383)	0.750
20 years old or older	2.233 (1.864, 2.676)	<0.001	1.992 (1.653, 2.401)	<0.001
(9) Practice sustainable dietary habits				
Elementary school ages or younger	1.264 (0.990, 1.614)	0.059	1.458 (1.136, 1.871)	0.003
Junior-high-school ages or older	1.505 (0.989, 2.290)	0.056	1.561 (1.025, 2.377)	0.038
20 years old or older	1.891 (1.429, 2.501)	<0.001	1.678 (1.262, 2.231)	<0.001
(10) Develop your understanding of food				
Elementary school ages or younger	1.027 (0.860, 1.227)	0.763	1.075 (0.894, 1.294)	0.437
Junior-high-school ages or older	1.278 (0.974, 1.678)	0.076	1.309 (0.992, 1.727)	0.057
20 years old or older	1.642 (1.365, 1.976)	<0.001	1.505 (1.242, 1.822)	<0.001
Adherence to a Japanese-style diet				
Elementary school ages or younger	1.361 (1.129, 1.641)	0.001	1.642 (1.345, 2.006)	<0.001
Junior-high-school ages or older	0.907 (0.682, 1.206)	0.504	1.014 (0.751, 1.368)	0.926
20 years old or older	2.327 (1.895, 2.858)	<0.001	1.937 (1.561, 2.404)	<0.001
*Attitude and knowledge*				
Attitude to food and nutrition education				
Elementary school ages or younger	1.802 (1.406, 2.310)	<0.001	1.689 (1.305, 2.188)	<0.001
Junior-high-school ages or older	0.891 (0.614, 1.294)	0.546	0.950 (0.648, 1.394)	0.796
20 years old or older	2.598 (1.949, 3.461)	<0.001	2.349 (1.746, 3.160)	<0.001
Knowledge of Dietary Balance Guide				
Elementary school ages or younger	1.642 (1.362, 1.979)	<0.001	1.474 (1.208, 1.797)	<0.001
Junior-high-school ages or older	0.828 (0.627, 1.094)	0.186	0.856 (0.640, 1.147)	0.300
20 years old or older	1.628 (1.344, 1.971)	<0.001	1.583 (1.292, 1.939)	<0.001
Knowledge of dietary guidelines				
Elementary school ages or younger	1.193 (0.993, 1.433)	0.058	1.212 (1.003, 1.465)	0.046
Junior-high-school ages or older	1.307 (0.999, 1.710)	0.050	1.276 (0.972, 1.675)	0.078
20 years old or older	1.450 (1.208, 1.742)	<0.001	1.428 (1.183, 1.724)	<0.001
Knowledge of Japanese-style diet				
Elementary school ages or younger	1.076 (0.900, 1.285)	0.418	1.247 (1.037, 1.501)	0.019
Junior-high-school ages or older	1.467 (1.125, 1.912)	0.005	1.405 (1.072, 1.840)	0.013
20 years old or older	1.445 (1.208, 1.729)	<0.001	1.385 (1.150, 1.668)	0.001

The life stage of food production experience. Without experience: n = 2187 [ref.], with experience during elementary school ages or younger: n = 798, junior-high-school ages or older: n = 304, and 20 years old or older: n = 624 (multiple responses). B: regression coefficient (linear regression analysis); OR: odds ratio (logistic regression analysis); CI: confidence interval. * 13–52 points: the lower the score, the higher the adherence. ** (1) Enjoy your meals; (2) establish a healthy rhythm by establishing regular times for meals; (3) maintain appropriate weight through adequate exercise and well-balanced meals; (4) eat well-balanced meals with staple food, as well as main and side dishes; (5) eat enough grains, such as rice and other cereals; (6a) ensure vitamin, mineral, and fiber intakes through consumption of vegetables and fruit on a daily basis; (6b) maintain calcium intake from milk, dairy products, green and yellow vegetables, beans, and small fish; (7a) avoid food and dishes with a high salt content; (7b) consume a balanced intake of fats from animals, vegetables, and fish; (7c) establish a habit of checking nutrition fact labels when selecting food and eating out; (8) take advantage of the Japanese dietary culture and local food products, and preserve local dishes; (9) conserve food resources and practice dietary habits to minimize leftovers and food waste; and (10) develop your understanding of food, and review your dietary life.

**Table 4:** Life stage at which food production experience occurred and behavior, attitude, and knowledge of dietary recommendations in their 50s and 60s. (N = 1409).

	Crude Models		Adjusted Models	
	B (95%CI)	*p*	B (95%CI)	*p*
*Behavior*				
Total score for dietary guideline adherence *				
Elementary school age or younger	−0.777 (−1.843, 0.287)	0.153	−0.756 (−1.774, 0.260)	0.145
Junior-high-school ages or older	−0.278 (−1.734, 1.178)	0.708	−0.760 (−2.152, 0.630)	0.284
20 years or older	−1.764 (−2.562, −0.967)	<0.001	−1.314 (−2.082, −0.546)	<0.001
Each item of dietary guidelines adherence **				
(1) Enjoy your meals	OR (95%CI)		OR (95%CI)	
Elementary school ages or younger	1.238 (0.768, 1.996)	0.379	1.272 (0.782, 2.070)	0.331
Junior-high-school ages or older	1.626 (0.782, 3.380)	0.192	1.980 (0.943, 4.156)	0.070
20 years old or older	1.366 (0.953, 1.958)	0.088	1.191 (0.818, 1.733)	0.360
(2) Establish a healthy dietary rhythm				
Elementary school ages or younger	0.786 (0.524, 1.180)	0.247	0.769 (0.509, 1.162)	0.213
Junior-high-school ages or older	1.018 (0.575, 1.802)	0.948	1.139 (0.638, 2.032)	0.658
20 years old or older	1.822 (1.292, 2.571)	<0.001	1.644 (1.157, 2.336)	0.005
(3) Maintain appropriate body weight				
Elementary school ages or younger	1.023 (0.705, 1.486)	0.901	1.023 (0.702, 1.489)	0.905
Junior-high-school ages or older	1.364 (0.805, 2.313)	0.248	1.405 (0.826, 2.389)	0.209
20 years old or older	1.156 (0.872, 1.531)	0.312	1.105 (0.830, 1.472)	0.490
(4) Eat a well-balanced meal				
Elementary school ages or younger	1.157 (0.753, 1.780)	0.504	1.154 (0.742, 1.794)	0.524
Junior-high-school ages or older	0.833 (0.465, 1.492)	0.539	0.968 (0.531, 1.763)	0.916
20 years old or older	1.768 (1.255, 2.490)	0.001	1.552 (1.089, 2.213)	0.015
(5) Eat enough grains				
Elementary school ages or younger	0.921 (0.589, 1.439)	0.719	0.900 (0.574, 1.412)	0.648
Junior-high-school ages or older	1.697 (0.852, 3.377)	0.131	1.775 (0.889, 3.544)	0.103
20 years old or older	1.248 (0.878, 1.773)	0.215	1.196 (0.838, 1.707)	0.323
(6a) Eat enough fruit and vegetable				
Elementary school ages or younger	1.360 (0.907, 2.040)	0.136	1.353 (0.896, 2.044)	0.149
Junior-high-school ages or older	1.024 (0.586, 1.789)	0.931	1.186 (0.670, 2.099)	0.557
20 years old or older	1.323 (0.980, 1.785)	0.066	1.211 (0.889, 1.650)	0.223
(6b) Maintain calcium intake				
Elementary school ages or younger	1.039 (0.693, 1.558)	0.850	1.030 (0.685, 1.549)	0.884
Junior-high-school ages or older	0.891 (0.515, 1.540)	0.679	0.985 (0.567, 1.713)	0.959
20 years old or older	1.146 (0.844, 1.557)	0.379	1.074 (0.785, 1.468)	0.652
(7a) Reduce salt intake				
Elementary school ages or younger	1.044 (0.722, 1.511)	0.815	1.077 (0.741, 1.567)	0.694
Junior-high-school ages or older	1.172 (0.701, 1.960)	0.543	1.265 (0.752, 2.129)	0.375
20 years old or older	1.346 (1.017, 1.782)	0.037	1.286 (0.964, 1.715)	0.086
(7b) Consume fats appropriately				
Elementary school ages or younger	0.968 (0.663, 1.413)	0.867	0.947 (0.645, 1.389)	0.781
Junior-high-school ages or older	1.187 (0.698, 2.019)	0.526	1.318 (0.768, 2.262)	0.315
20 years old or older	1.470 (1.096, 1.972)	0.010	1.361 (1.008, 1.836)	0.043
(7c) Use nutrition fact labels				
Elementary school ages or younger	1.130 (0.770, 1.657)	0.530	1.145 (0.772, 1.698)	0.498
Junior-high-school ages or older	1.123 (0.671, 1.880)	0.656	1.240 (0.730, 2.105)	0.425
20 years old or older	1.455 (1.098, 1.929)	0.009	1.346 (1.007, 1.800)	0.044
(8) Preserve traditional diets				
Elementary school ages or younger	1.368 (0.952, 1.965)	0.089	1.370 (0.946, 1.982)	0.094
Junior-high-school ages or older	0.976 (0.593, 1.606)	0.925	1.091 (0.655, 1.816)	0.735
20 years old or older	2.149 (1.639, 2.817)	<0.001	1.995 (1.511, 2.633)	<0.001
(9) Practice sustainable dietary habits				
Elementary school ages or younger	0.979 (0.569, 1.684)	0.940	1.022 (0.594, 1.759)	0.936
Junior-high-school ages or older	2.561 (0.942, 6.965)	0.065	2.652 (0.977, 7.202)	0.055
20 years old or older	1.933 (1.205, 3.102)	0.006	1.913 (1.187, 3.083)	0.007
(10) Develop your understanding of food				
Elementary school ages or younger	1.222 (0.842, 1.775)	0.289	1.235 (0.845, 1.805)	0.275
Junior-high-school ages or older	1.475 (0.864, 2.517)	0.153	1.681 (0.976, 2.894)	0.061
20 years old or older	1.549 (1.168, 2.056)	0.002	1.419 (1.061, 1.897)	0.018
Adherence to a Japanese-style diet				
Elementary school ages or younger	1.424 (0.940, 2.157)	0.094	1.506 (0.983, 2.306)	0.059
Junior-high-school ages or older	1.609 (0.858, 3.016)	0.137	1.898 (0.997, 3.613)	0.050
20 years old or older	2.170 (1.565, 3.008)	<0.001	2.039 (1.454, 2.861)	<0.001
*Attitude and knowledge*				
Attitude to food and nutrition education				
Elementary school ages or younger	3.081 (1.732, 5.482)	<0.001	3.188 (1.787, 5.688)	<0.001
Junior-high-school ages or older	0.726 (0.352, 1.496)	0.386	0.855 (0.410, 1.785)	0.678
20 years old or older	2.529 (1.697, 3.768)	<0.001	2.218 (1.472, 3.343)	<0.001
Knowledge of Dietary Balance Guide				
Elementary school ages or younger	1.598 (1.100, 2.321)	0.013	1.681 (1.144, 2.470)	0.008
Junior-high-school ages or older	0.732 (0.442, 1.214)	0.228	0.806 (0.478, 1.361)	0.421
20 years old or older	1.666 (1.263, 2.198)	<0.001	1.543 (1.156, 2.059)	0.003
Knowledge of dietary guidelines				
Elementary school ages or younger	1.086 (0.751, 1.571)	0.659	1.069 (0.734, 1.558)	0.725
Junior-high-school ages or older	1.487 (0.907, 2.440)	0.115	1.617 (0.977, 2.676)	0.061
20 years old or older	1.552 (1.183, 2.037)	0.001	1.469 (1.112, 1.941)	0.006
Knowledge of Japanese-style diet				
Elementary school ages or younger	1.290 (0.900, 1.849)	0.164	1.271 (0.885, 1.825)	0.192
Junior-high-school ages or older	1.663 (1.000, 2.763)	0.049	1.711 (1.027, 2.851)	0.038
20 years old or older	1.285 (0.981, 1.682)	0.068	1.228 (0.934, 1.614)	0.139

The life stage of food production experience. Without experience: n = 957 [ref.], with experience during elementary school ages or younger: n = 203, junior-high-school ages or older: n = 101, and 20 years old or older: n = 291 (multiple responses). B: regression coefficient (linear regression analysis); OR: odds ratio (logistic regression analysis); CI: confidence interval. * 13–52 points: the lower the score, the higher the adherence. ** (1) Enjoy your meals; (2) establish a healthy rhythm by establishing regular times for meals; (3) maintain appropriate weight through adequate exercise and well-balanced meals; (4) eat well-balanced meals with staple food, as well as main and side dishes; (5) eat enough grains, such as rice and other cereals; (6a) ensure vitamin, mineral, and fiber intakes by consumption of vegetables and fruit on a daily basis; (6b) maintain calcium intake from milk, dairy products, green and yellow vegetables, beans, and small fish; (7a) avoid food and dishes with a high salt content; (7b) consume a balanced intake of fats from animals, vegetables, and fish; (7c) establish a habit of checking nutrition fact labels when selecting food and eating out; (8) take advantage of the Japanese dietary culture and local food products, and preserve local dishes; (9) conserve food resources and practice dietary habits to minimize leftovers and food waste; and (10) develop your understanding of food and review your dietary life.

## Data Availability

It is available by applying to the Social Science Japan Data Archive, Center for Social Research and Data Archive, which is affiliated with the Institute of Social Sciences, University of Tokyo.
